# Soil fungal community development in a high Arctic glacier foreland follows a directional replacement model, with a mid-successional diversity maximum

**DOI:** 10.1038/srep26360

**Published:** 2016-05-31

**Authors:** Ke Dong, Binu Tripathi, Itumeleng Moroenyane, Woosung Kim, Nan Li, Haiyan Chu, Jonathan Adams

**Affiliations:** 1 Department of Biological Sciences, College of Natural Sciences, Seoul National University, Seoul, 08826, South Korea; 2 Arctic Research Centre, Korea Polar Research Institute, Incheon, 21990, South Korea; 3 Division of Life Sciences, School of Science, The Hong Kong University of Science and Technology, Clear Water Bay, Kowloon, 999077, Hong Kong; 4 Institut National de la Recherche Scientifique, Centre INRS-Institut Armand-Frappier, 531 boulevard de Prairies, Laval, Quebec, H7V 1B7, Canada; 5 School of the Environment, Florida A&M University, Tallahassee, FL, 32307, USA; 6 State Key Laboratory of Soil and Sustainable Agriculture, Institute of Soil Science, Chinese Academy of Sciences, East Beijing Road 71, Nanjing 210008 China

## Abstract

Directional replacement and directional non-replacement models are two alternative paradigms for community development in primary successional environments. The first model emphasizes turnover in species between early and late successional niches. The second emphasizes accumulation of additional diversity over time. To test whether the development of soil fungal communities in the foreland of an Arctic glacier conforms to either of these models, we collected samples from the Midtre Lovénbreen Glacier, Svalbard, along a soil successional series spanning >80 years. Soil DNA was extracted, and fungal ITS1 region was amplified and sequenced on an Illumina Miseq. There was a progressive change in community composition in the soil fungal community, with greatest fungal OTU richness in the Mid Stage (50–80 years). A nestedness analysis showed that the Early Stage (20–50 years) and the Late Stage (>80 years) fungal communities were nested within the Mid Stage communities. These results imply that fungal community development in this glacier succession follows a directional replacement model. Soil development processes may initially be important in facilitating arrival of additional fungal species, to give a mid-successional diversity maximum that contains both early- and late-successional fungi. Competition may then decrease the overall diversity due to the loss of early successional species.

The processes by which communities and ecosystems assemble themselves in new or disturbed environments have long fascinated ecologists^1^. One of the most frequently studied successional environments is the foreland area uncovered by retreating glaciers^2^,^3^. Different models of community assembly have been tested and observed in primary succession^4^,^5^,^6^. During the 20th Century, two different models describing primary vegetation succession (directional replacement and directional non-replacement) were proposed and modified^7^,^8^. These two models have also been applied to invertebrate and fungi^9^,^10^. The dominant model of primary vegetation succession is essentially the directional replacement model, which emphasizes the replacement of primary colonizers by later stage species, and with a dominant role of competition in driving this community change^11^. On the other hand, invertebrate succession in glacier forelands has been found to follow a directional non-replacement model - with the progressive addition and persistence of taxa, and little or no loss of species over time^10^.

A number of studies have examined fungal communities from forelands of retreating glaciers, and their results revealed that time since deglaciation has large effects on fungal community composition^9^,^12^,^13^,^14^,^15^. A recent study of the Midtre Lovénbreen glacier on Svalbard found that primary succession of root-associated fungi of the ectomycorrhizal forb *Bistorta vivipara* (L.) Delabre (Polygonaceae) exhibited a ‘directional non-replacement model’ pattern of community change in following its host plant^9^. However, the root-associated fungal community alone is not necessarily representative of soil fungi in general. Root-associated fungi are always strongly influenced by their host plant, and so this community is particularly strongly tied to the species composition, ground cover and activity of vegetation^9^,^16^.

Soil fungal communities in successional systems are complex and they interact with many factors, beyond just the composition of the aboveground vegetation^17^,^18^,^19^,^20^. For instance, the buildup of organic nutrients like soil carbon and nitrogen derived from primary plant colonizers is considered to be one important aspect of soil fungal community development as the density of primary plant colonizers increases in a successional system^21^. Stochastic factors depending on colonization of soil organisms can also strongly influence soil fungal communities in successional systems. For example, Jumpponen^2^ noted the importance of airborne spore deposition in the fungal communities. While the aerially deposited, dormant spore bank is present across the succession, it is masked in the older substrate areas by a larger active mycelial biomass. Therefore, the whole fungal community of the bulk soil of a glacial foreland would not be expected to follow strictly the same successional patterns as root-associated fungi.

We were interested in understanding the fungal communities of the Midtre Lovénbreen glacier foreland on Svalbard, against the background of the previously published study of root-associated fungi at the same site by Davey *et al.*^9^, to test whether soil fungal community variation along the chronosequence also follows the directional non-replacement model. However, our primary hypothesis was that the directional replacement model would instead be the best description of overall soil fungal community change, based on the following mechanisms: 1) changes in soil conditions such as pH and nutrient concentrations, as the glacier foreland soil develops over several decades, should favour different sets of fungal species in terms of physiological requirements. This will affect their viability in competition, causing turnover of species. 2) During the early stages of glacier foreland soil development, soil resources are patchily distributed, and the soil fungal community is dominated by opportunistic lottery competition^22^ which favours rapid colonization and population growth on resource patches relatively free of other competing species. As the ecosystem develops over time, soil resources will build up and become less patchily distributed: lottery competition becomes less important, and steady competition between species and their competitive hierarchy under this form of competition begins to play an increasing role in the community^23^. This, we predicted, would eliminate fungal species that are generally weak competitors when placed under conditions of steady competition, causing loss of these species and dominance of communities by other species (which may have been slow to arrive and colonize) that are strong competitors. We also hypothesized that the soil fungal succession would relate strongly to soil total organic carbon (TOC) and total nitrogen (TN), as is the case with glacial succession generally for other groups of organisms and in glacier forelands elsewhere^21^,^24^.

## Results

### Fungal community analysis

A total of 161,200 high quality ITS1 region sequences were assigned to 774 OTUs at ≥97% similarity level from 31 samples after the removal of low quality, chimeric, and rare sequences. On average, 165 OTUs (±7.1 SE) were found in each sample. The individual highest OTU abundance was found in the Mid Stage with 406 OTUs, while the lowest OTU abundance was found in the Early Stage with 134 OTUs. The fungal communities in the deglaciated foreland area were dominated by Ascomycota which accounted for 79.2% of the classifiable reads, followed by Basidiomycota (5.5%), Zygomycota (4.1%) and Chytridiomycota (0.2%) or as basal fungal lineages. Basidiomycota and Chytridiomycota showed significant lower abundance in the Early Stage than Mid Stage and Late Stage (p < 0.05). A total of 4,636 sequences belonged to known groups of EcM fungi, representing 2.9% of the subsampled sequences. 11 genera were identified belonging to possible EcM fungi from our soil samples, with *Inocybe* as the most dominant genus (36.9% of total EcM sequences), followed by *Cortinarius* (28.8%).

Six OTUs had an abundance of more than 2% of the total sequences, and four of these (OTU00026, OTU00034, OTU00037, and OTU00046, all classified as Ascomycota) showed a significant or marginally significant difference between the Early Stage and the Late Stage (p ≤ 0.05). A total of 77 OTUs (18.5% of all generated reads) were potential indicator OTUs. Though the database cannot classify all of the sequences at a high taxonomic resolution, at a broad level most of these OTUs (63.6% of OTUs) were classified as Ascomycota. Basidiomycota as indicators were found only in the Late Stage. Among the indicator OTUs, 40 (12.6% of reads) were from the Early Stage; 18 (2.8% of reads) were from the Mid Stage and 21 (3.1% of reads) were from the Late Stage (Supplementary Table 1).

**Figure 1:**
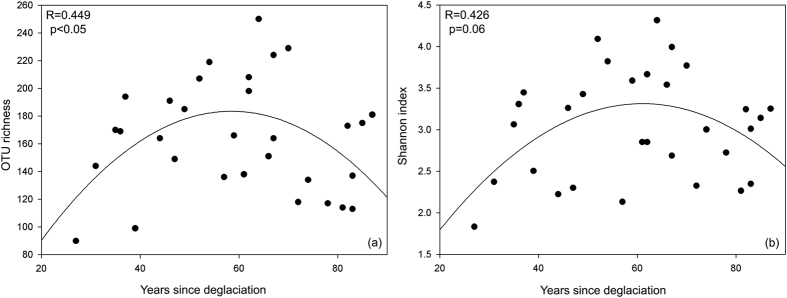
Fungal diversity measured along the chronosequence. (**a**) OTU richness and (**b**) Shannon index were used. To assess the relationship of years since deglaciation with diversity/richness indices of the total fungal community, we fitted three regression models (linear, quadratic, and cubic). Model selection was carried out based on adjusted R^2^ and root mean square error. Only the best fit quadratic regression was shown.

With increasing age since deglaciation, the fungal diversity showed a significant quadratic trend in relation to OTU richness (R = 0.449, p < 0.05) and a marginally significant quadratic regression by Shannon index (R = 0.426, p = 0.06) (Fig. 1). This revealed that OTU richness/diversity increased from the Early Stage of glacial succession to the Mid Stage, and then decreased from the Mid to Late Stage. Rarefaction curves of OTU richness also strongly supported the view that the Mid Stage had greater fungal diversity, with OTU richness per individual sample significantly greater than corresponding Early Stage and Late Stage samples (Supplementary Fig. S2). ANOSIM performed on Bray-Curtis community matrix revealed that samples from three different successional age categories varied significantly from each other (Table 1).

**Figure 2:**
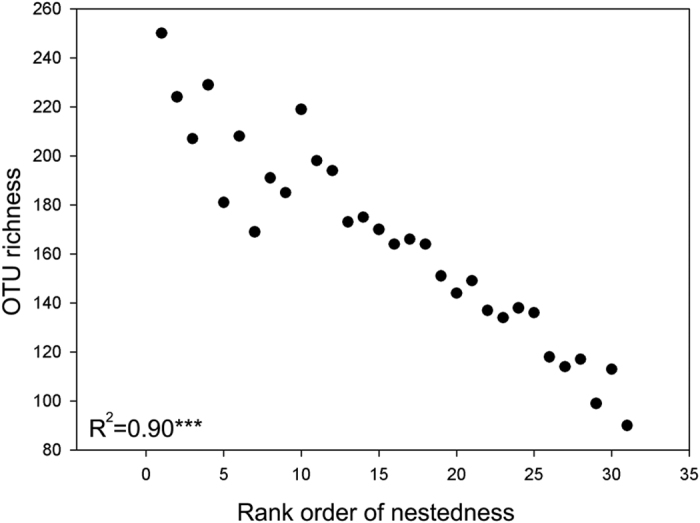
Soil fungal OTU richness re-ordered along the rank order of nestedness. Significance level is shown at ***p < 0.001.

Nestedness analysis showed that the fungal communities across the whole time span of succession follow a nested structure (P ≪ 0.0001). A packed matrix order of all samples was generated, in which the nestedness of each sample was categorized from high to low, and the lower ones are nested in the higher ones (Supplementary Table 4). The Spearman’s rho test indicated that this packed matrix order is highly correlated to the OTU richness of each sample (Fig. 2). Moreover, the OTU richness was found to be higher in the Mid Stage than in the Early Stage and Late Stage. All these results together indicate that those samples with lower OTU richness, which are mostly from Early Stage and Late Stage, tend to have an OTU composition that is a subset of Mid Stage communities.

### Physicochemical characteristics of the glacier foreland

**Figure 3:**
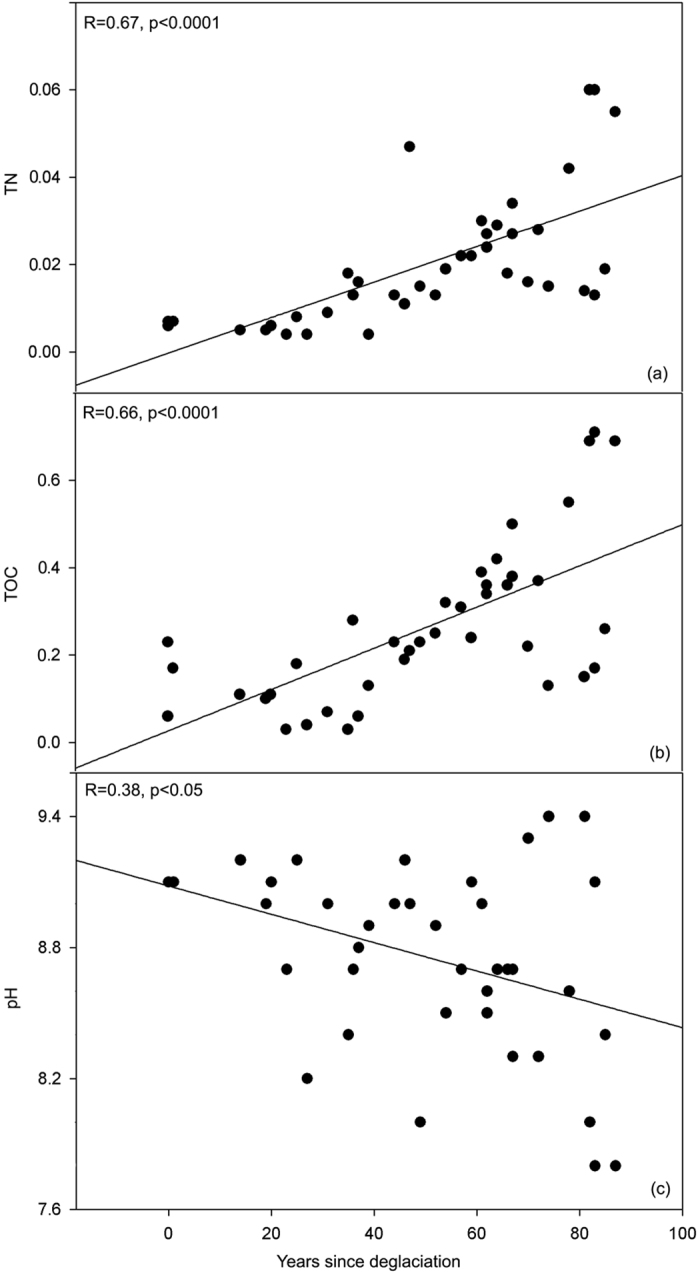
Three environmental parameters are shown along the age since deglaciation of soil in the glacier of Midtre Lovénbreen: (**a**) TN; (**b**) TOC; (**c**) pH. TOC, total organic carbon; TN, total nitrogen. The values of TOC and TN are shown in percentages.

With age since deglaciation, concentrations of important nutrients for microbial activity such as TOC and TN increased steadily, while pH decreased (Fig. 3). The TOC in the soil increased significantly from a very low content of 0.03% in the newly exposed soils, which are close to the edge of glacier, to over 0.69% in the oldest foreland soils around 80 years old (R = 0.66, p < 0.0001), while the content of TN increased from 0.004% to 0.055% (R = 0.67, p < 0.0001). In contrast, the pH - which ranged from 7.8 to 9.4 in the whole sample set - decreased along the chronosequence (R = 0.38, p < 0.05). No significant time-related patterns were found in TP, C/N ratio, soil moisture, and sand or clay-silt proportion in relation to age since deglaciation. Cluster analysis of environmental variables and age since deglaciation in the retreating glacier area showed high correlation between TOC and TN (Spearman ρ^2^ ≥ 0.6; Supplementary Fig. S3), suggesting TOC and TN are covariables.

**Figure 4:**
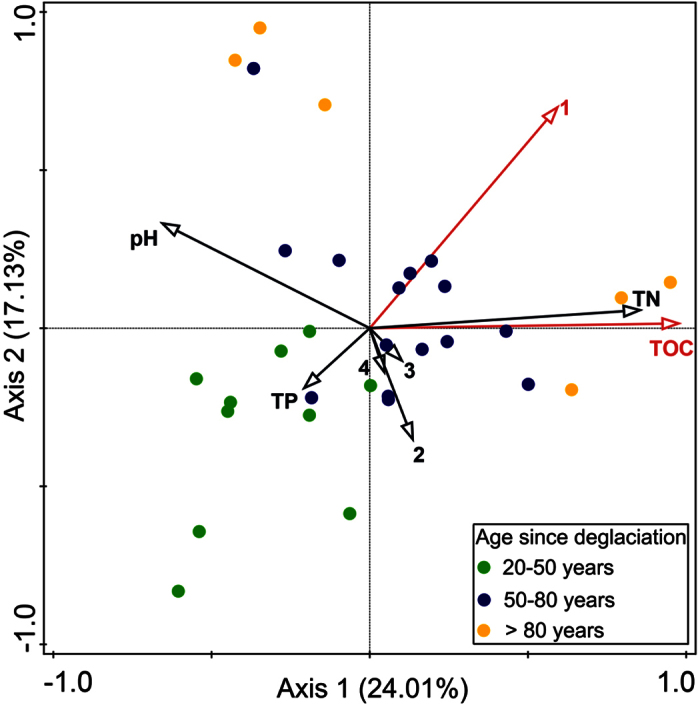
Canonical Correspondence Analysis ordination plot of fungal community composition based on ITS1 gene OTUs and a vector overlay of the environmental variables. Environmental variables were shown in arrows and the significant ones were presented in red. Different colors of dots denote different age since deglaciation: green, 20–50 years; blue, 50–80 years; yellow, over 80 years. 1, age since deglaciation; 2, soil moisture; 3, C/N ratio; 4, proportion of silt and clay; TN, total nitrogen; TOC, total organic carbon; TP, total phosphorus.

CCA analysis was used to reveal which environmental properties best predicted variation in fungal communities in the deglaciated foreland area (Fig. 4). Together with two axes on the biplot, in an accumulative variance for the interaction between communities and variables, a total of 41.1% of variation was explained. Axis 1 explained 24.0% of the variation in the data, while axis 2 explained 17.1%. Axis 1 and 2 together separated out the three stages, with Early Stage soils associated with low values of CCA axis 1 and 2. TOC, pH, C/N ratio, soil moisture, the proportion of silt and clay, age since deglaciation, TN and TP together accounted for 30.6% of the total fungal community variation in combination. Among these selected environmental factors, TOC (pseudo-F = 2.2, p < 0.001) and deglaciated age (pseudo-F = 1.6, p < 0.005) were significant contributors to fungal community variability, and a forward test indicated that the most important factor was TOC.

## Discussion

We expected to see a directional replacement trend in community composition during succession, under the hypothesis that competition between species would begin to play an increasing role as nutrients accumulated within the initially exposed low-nutrient soil from the glacier, and as total population densities increased. The result showed a significant peak in OTU richness/diversity in the Mid Stage successional soils, and a progressive change in community composition with increasing age (Fig. 4). From this observation, together with the fact that there is nesting of the relatively OTU-poor Early and Late Stage communities into Mid Stage OTU-rich communities, it appears that a directional replacement model is the most consistent model for changes in the soil fungal community across the whole successional time span of this glacier foreland. By contrast, other studies in high resistance environments typical of the High Arctic have found directional non-replacement succession in arbuscular and root-associated fungal communities^25^,^26^. For example, Davey *et al.* studied a relatively restricted system - the plant roots of one particular species of plant (*Bistoria*), in a system dominated by mutualistic interaction between the fungus and host plant^9^. In this sense, it is not surprising that the root-associated fungal community and the general soil fungal community may be following different trends. For example, the trade-offs for the host plant in terms of accumulating a diverse EcM community, as opposed to eliminating part of it, may need to be considered as part of the process^27^. This consideration might go some way to explaining why EcM diversity instead showed turnover in another more fertile environment, an alpine glacier foreland^9^. Accumulating extra EcM species as they arrive in a relatively resource poor environment may improve the flexibility of nutrient capture by the host plant^27^. In contrast, in a more nutrient-rich alpine glacier foreland, the same plant species may be more selective about its fungal community, eliminating some species which are no longer helpful in resource capture^9^. Additionally, from the perspective of fungal-fungal interaction, the competition between fungal species might be intense in a more nutrient rich environment (such as an alpine glacier foreland), which may also play a role in eliminating some species.

In this study, Ascomycota indicator OTUs occurred most frequently in the Early Stage of succession, while Basidiomycota indicator OTUs were more frequent in the Late Stage. Four of the six most abundant OTUs in the whole dataset were classified as Ascomycota and each showed a decreasing trend with deglaciated age. These results, and the change in the whole community composition (Fig. 4), suggest a replacement of Ascomycota by Basidiomycota along the chronosequence. Elsewhere, similar turnover in the fungal community, with Ascomycota replaced by Basidiomycota during succession, has been reported by Zumsteg *et al.*^21^ in the Damma Glacier foreland in the Central Alps. It is known that many groups of Ascomycota are abundant in extreme environments^28^,^29^,^30^ for example *Lecanoromycetes*, to which most of the lichen-forming fungal species belong^29^, and *Dothidomycetes* which is the ‘pioneer’ in unfavorable environments and enhances plant growth by improving nutrient and water acquisition^28^. By contrast, Basidiomycota colonize more heavily vegetated soils^21^. The trend of Ascomycota being replaced by Basidiomycota along the succession therefore may imply: i) the accumulation of nutrients, ii) the maturation of the ecosystem. We were interested in finding what specific fungal subgroups of the Ascomycota and Basidiomycota gave rise to the turnover during succession, but unfortunately low resolution of our ITS1 sequences in the available databases results prevented interpretation at levels below phylum, in many cases. Lack of taxonomic resolution also hampered our identification of EcM fungi. However, the relative abundance of EcM sequences showed a linear regression with the deglaciated age (R = 0.43, p < 0.05, Supplementary Fig. 1) and was significantly lower in the early stage than middle stage and late stage (P < 0.05).

TOC, TN and pH, which showed high correlation to the deglaciated age in this study, have been considered important soil properties in shaping fungal communities^24^,^31^. However, only TOC, besides the deglaciated age, was selected as a significant influencing variable by the forward selection in CCA analysis. TOC and TN were classified as covariables by a cluster analysis. As these two factors emerge as being so close in terms of predictive power, is difficult to tell whether TOC or TN is actually more important. However, considering that fungi are heterotrophic and that some fungi can fix nitrogen^32^, it seems less unlikely that nitrogen limitation is the strongest structuring factor for the community because they are not wholly dependent on soil nitrogen. We therefore would suggest that TOC is a more important environmental variable in primary glacier succession.

In the succession we observed here, fungal OTU richness/diversity increased from the Early Stage towards the Mid Stage, and declined from the Mid Stage to the Late Stage. We suggest that the later decline in diversity might partly occur because of increasing importance of steady interference competition (as opposed to very variable lottery competition^22^ on highly localized resource patches) over time as both fungal population densities and TOC and TN build up in the soil. The initial increase in diversity from the Early Stage to Mid Stage demands other explanations. In the Early Stage, diversity may be low because fewer viable niches are available in the very resource-poor soil, before significant build-up of TOC and TN. In these circumstances, the ability to colonize very patchy localized resources within the soil matrix, the ability to continue to grow slowly under low resource availability, and the physiological adaptations to higher soil pH, may all be necessary for establishment and survival. These would generally be regarded in ecology as conditions in which interspecific competitive interactions were weak and/or unpredictable, although the use of the term ‘competition intensity’ is slippery and elusive^23^.

As resources in the soil accumulate with soil development^33^,^34^ and the pH becomes closer to neutral, the physiological specializations of the pioneer species may no longer be generally necessary, and a greater range of fungal species will be able to survive^35^. Furthermore, there has now been time for groups that are slow dispersers but strong competitors to arrive^36^. Nevertheless, we suggest that by the Mid Stage the resource concentrations are still low enough, or patchy enough, that many of the earlier pioneer species can still coexist along with the new arrivals (as is shown by the nestedness analysis, where the ‘Early Stage’ community is shown to be contained within the ‘Mid Stage community’). A key factor may be that the fine scale mosaic of environments to some extent shields the pioneer fungal species against intense interference competition. This would be analogous to the situation in plant communities many mid successional environments, where ‘pioneer’ plant species coexist for a time in a fine scale mosaic of low soil nutrient conditions alongside faster-growing later successional species that require higher nutrient concentrations^37^.

By the Late Stage, as a result of several decades of plant growth and decomposition, high resource concentrations are predictable enough throughout the soil that population densities of fungi are higher, with intense and stable interference competition and less opportunity for ‘lottery’ colonization of localized resources within the soil matrix. Fungi that can most effectively exploit these more abundant, stable resources are able to dominate, at the expense of the ‘pioneer’ fungi that had persisted into the Mid Stage. The loss of these early-stage fungi causes a decrease in overall diversity. This would be analogous to the displacement of ‘stress tolerant’ pioneer vascular plants in primary successional systems as conditions are ameliorated^11^,^38^.

A second and slightly different explanation may be that rather than the changing intensity of ‘competition’ being important in producing the pattern of community change and diversity, there is simply a distinct set of ‘early’ successional niches and another set of ‘late’ successional niches^39^,^40^. The ‘mid-stage’ diversity maximum may thus reflect a stage of intermediate soil development, pH and nutrient concentrations, in which both ‘early’ and ‘late’ niches are viable in competition. The result of this overlap is greater diversity. As soil develops beyond the niche limits of early successional species, they are lost and only late successional species survive (under lower diversity).

It would be very interesting to study other successional systems along chronosequences, to understand how widespread the turnover patterns we observed here are in fungal ecology. Additionally, field-based or laboratory-based microcosm systems might be able to add far more detailed observations, and tests of mechanisms that can come from experimental manipulation of conditions. Combining the metagenetic approach used here with metagenetic sampling of small soil metazoans, bacteria and archaea, and also adding soil metagenomes, could potentially give far more detailed and sophisticated understanding of the mechanisms in community and ecosystem development of primary successional systems.

## Methods

### Sampling and site description

Our sampling site was the foreland of the retreating Midtre Lovénbreen Glacier, Svalbard (Supplementary Table 2). The glacier began to retreat in the 1920s, leaving an area of moraine which now covers around 10 km^2^. Progressive time stages of the retreat have been mapped based on high resolution aerial photography and satellite imagery^41^. Interpolation between these isochrones allows estimation of the age since deglaciation of each point in the foreland. Moreau *et al.*^41^ described the vegetation in this deglaciated area as strongly influenced by age since deglaciation, and further from successional equilibrium than tundra outside the glacial foreland zone. In the foreland, pioneer species including *Salix polaris* are found as a sparse vegetation in younger areas, while other species such as *Carex nardina* and *Polygonum viviparum* also occur in the older foreland soils.

We took three transects, running from the current snout of the glacier to the edge of the moraine that has formed since the 1920s (Supplementary Figs S4–S6). In total, 39 samples were collected from three transects from the glacier front to the edge of the foreland moraine to ensure a representative coverage of the foreland (15 samples were taken from transect 1, 15 from transect 2, and 9 from transect 3). Because of the differing distances covered during retreat in different parts of the foreland, Transect 1 was sampled every 50 meters, and the Transect 2 was sampled every 80 meters, reflecting the longer length of the middle part of the foreland due to more rapid glacial recession. Transect 3 was sampled near transect 2 to ensure that enough samples were collected from each soil stage. At each sampling site, five subsamples were taken within a 1 m^2^ quadrat, taking a scoop of 100 g of soil at 0–10 cm depth at each corner and at the central point of the square. The five subsamples were pooled together to make one sample of 500 g for that 1 m^2^ quadrat. Sampling was carried out over 3 days at the end of July and beginning of August 2014.

### DNA extraction and soil properties

On the same day as sampling, soil DNA extraction and soil moisture measurement were carried out at the field station laboratory on Svalbard. The remainder of each soil sample was packed and stored at −20 °C. The soil DNA was extracted using the MoBio Power Soil DNA isolation kit (MoBio Laboratories, Carlsbad, CA, USA) following manufacturer’s instructions, and random empty vials were chosen and run to serve as controls. The isolated DNA was stored at −80 °C until the PCR stage. Soil texture, TOC, TN, total phosphorus (TP), and pH were measured at National Instrumentation Center for Environmental Management, Korea by following the standard protocol of the Soil Science Society of America (SSSA).

### PCR amplification and sequencing of ITS1 region

Each sequenced sample is prepared according to the Illumina 16S Metagenomic Sequencing Library protocols to amplify the ITS1 region. The DNA quality is measured by PicoGreen and Nanodrop. Input gDNA (10 ng) is PCR amplified. The barcoded fusion primer sequences used for amplifications were as follows: ITS1-F (5′-CTTGGTCATTTAGAGGAAGTAA-3′) and ITS2 (5′-GCTGCGTTCTTCATCGATGC-3′). The final purified product is then quantified using qPCR according to the qPCR Quantification Protocol Guide (KAPA Library Quantificatoin kits for Illumina Sequecing platforms) and the quality is identified by the LabChip GX HT DNA High Sensitivity Kit. (PerkinElmer, Massachusetts, USA). And then the paired-end (2 × 300 bp) sequencing was performed by the Macrogen Inc. (Seoul, Korea) using the MiSeq^TM^ platform (Illumina, San Diego, USA).

### Data analysis

Eight samples from the glacial foreland area were excluded from further analysis owing to either insufficient DNA for sequencing, or low read numbers (less than 2000 reads) generated by sequencing. Six of these eight excluded samples were collected from soils near the snout of the glacier (which had a deglaciated age less than 20 years). The remaining 31 samples were divided into three groups in relation to the isochrones of three main moraines^41^. The samples collected from areas deglaciated between 20 years and 50 years ago were categorized as the Early Stage. Those more than 50 years and less than 80 years old were assigned to the Mid Stage, while those more than 80 years old were assigned to the Late Stage.

The sequence data obtained was processed in Mothur^42^. Sequences with any ambiguous bases, sequences with more than 8 homopolymers and sequences with lengths less than 200 bp were removed using the screen.seqs command in Mothur. Putative chimeric sequences were detected and removed via the Chimera Uchime algorithm contained within Mothur^43^ in *de novo* mode, which first splits sequences into groups and then checks each sequence within a group using the more abundant groups as reference. Rare sequences (≤10 reads) were removed to avoid the risk of including spurious reads generated by sequencing errors^44^. A final even subsampling depth of 5,200 reads was applied to each sample. Operational taxonomic units (OTUs) were assigned using the QIIME implementation of UCLUST^45^, with a threshold of 97% pairwise identity. Taxonomic classification of each quality sequence was done against the UNITE version 6.0 database^46^ using the classify command in Mothur at an 80% cutoff with 1000 iterations. EcM fungi were determined by matching taxonomy assignments with established EcM lineages as determined by recent phylogenetic and stable isotope data^47^. The Miseq sequence data used in this study are deposited in the MG-RAST server^48^ under project ID 15403 (http://metagenomics.anl.gov/metagenomics.cgi?page=MetagenomeProject&project=15403).

### Statistical analysis

To assess the relationship between fungal richness/diversity and environmental variables, as well as with age since deglaciation, the richness of OTUs and other diversity indices were calculated using the Mothur platform^42^ for samples standardized to 5,200 reads (size decided by default, set to the size of smallest sample size), as diversity is directly correlated with the number of sequences collected. Bray-Curtis distance was calculated to analyze fungal community similarity. To reduce the contribution of highly abundant OTUs in relation to less abundant ones in the calculation of Bray-Curtis measure, abundance data of OTUs were square root transformed. The pairwise differences in community composition of the four groups were calculated by analysis of similarity (ANOSIM) in relation to Bray-Curtis distance. To assess the best fitting model of correlations between age since deglaciation and diversity/environmental variables, linear, polynomial (quadratic) and power (cubic) models were tried out using SigmaPlot v 10.0 (Systat Software, San Jose, CA). R version 2.15.2 and R package ‘labdsv’ was used to calculate the indicator value and the significance of OTUs^49^. Only OTUs with greater than 10% frequency, an indicator value >0.3^50^ and a p value <0.05 were selected as indicator OTUs. Cluster analysis of all measured environmental variables was carried out using the Varclus procedure in the Hmisc package in the R platform, version 2.15.2.

### Nestedness analysis and Canonical correspondence analysis

The nestedness analysis was performed by BINMATNEST with default input parameters^51^ to test the nested structure of the fungal communities. The significance of nestedness was tested using default input parameters and null model 3 which calculates the p-value for row and column totals following Geel *et al.*^52^. The correlation of OTU richness and packed matrix order (indicating nestedness categorized from high to low) of each sample was evaluated by Spearman’s rho test^53^. A constrained analysis was conducted using CANOCO^54^ to assess the effect of environmental variables on the fungal community. Canonical correspondence analysis (CCA)^55^ was used since the length of the extracted gradient was larger than 3 SD units. Forward selection was used to select significant explanatory variables with 999 permutations and only significant (p < 0.05) variables were included in the models.

## Additional Information

**How to cite this article**: Dong, K. *et al.* Soil fungal community development in a high Arctic glacier foreland follows a directional replacement model, with a mid-successional diversity maximum. *Sci. Rep.* **6**, 26360; doi: 10.1038/srep26360 (2016).

## Supplementary Material

Supplementary Information

## Figures and Tables

**Table 1:** R value and significance level (p) from 2-way ANOSIM test for differences in the fungal community structure between stages.

Groups	R statistic
ES, MS	0.226**
ES, LS	0.581***
MS, LS	0.274*

ES, Early Stage; MS, Mid Stage; LS, Late Stage. Significance codes: 0 ‘***’ 0.001 ‘**’ 0.01 ‘*’ 0.05 ‘.’ 0.1 ‘ ’ 1.
