# Supplementary material for: Soil fungal community development in a high Arctic glacier foreland follows a directional replacement model, with a mid-successional diversity maximum
Source: Sci Rep. 2016 May 31;6:26360. doi: 10.1038/srep26360 (PMC4886535; doi:10.1038/srep26360)
Supplement: Supplementary Information [file srep26360-s1.doc]

**Soil fungal community development in a high Arctic glacier foreland follows a directional replacement model, with a mid-successional diversity maximum**

Ke Dong^1^, Binu Tripathi^1^, Itumeleng Moroenyane^1,2^, Woosung Kim^1^, Nan Li^3^, Haiyan Chu^4^, Jonathan Adams^1,*^

1, Department of Biological Sciences, College of Natural Sciences, Seoul National University, Seoul 151-742, South Korea

2, Division of Life Sciences, School of Science, The Hong Kong University of Science and Technology, Clear Water Bay, Kowloon, 999077, Hong Kong

3, School of the Environment, Florida A&M University, Tallahassee, FL, 32307, USA

4, State Key Laboratory of Soil and Sustainable Agriculture, Institute of Soil Science, Chinese Academy of Sciences, East Beijing Road 71, Nanjing 210008 China

*Corresponding author: Jonathan Adams, Department of Biological Sciences, College of Natural Sciences, Seoul National University, Seoul 151-742, South Korea, (TEL) 82-2-880-4339, (Fax) 82-2-880-4962, (Email) [geograph.ecol@gmail.com](mailto:geograph.ecol@gmail.com)

Running title: Directional replacement model of arctic soil fungi


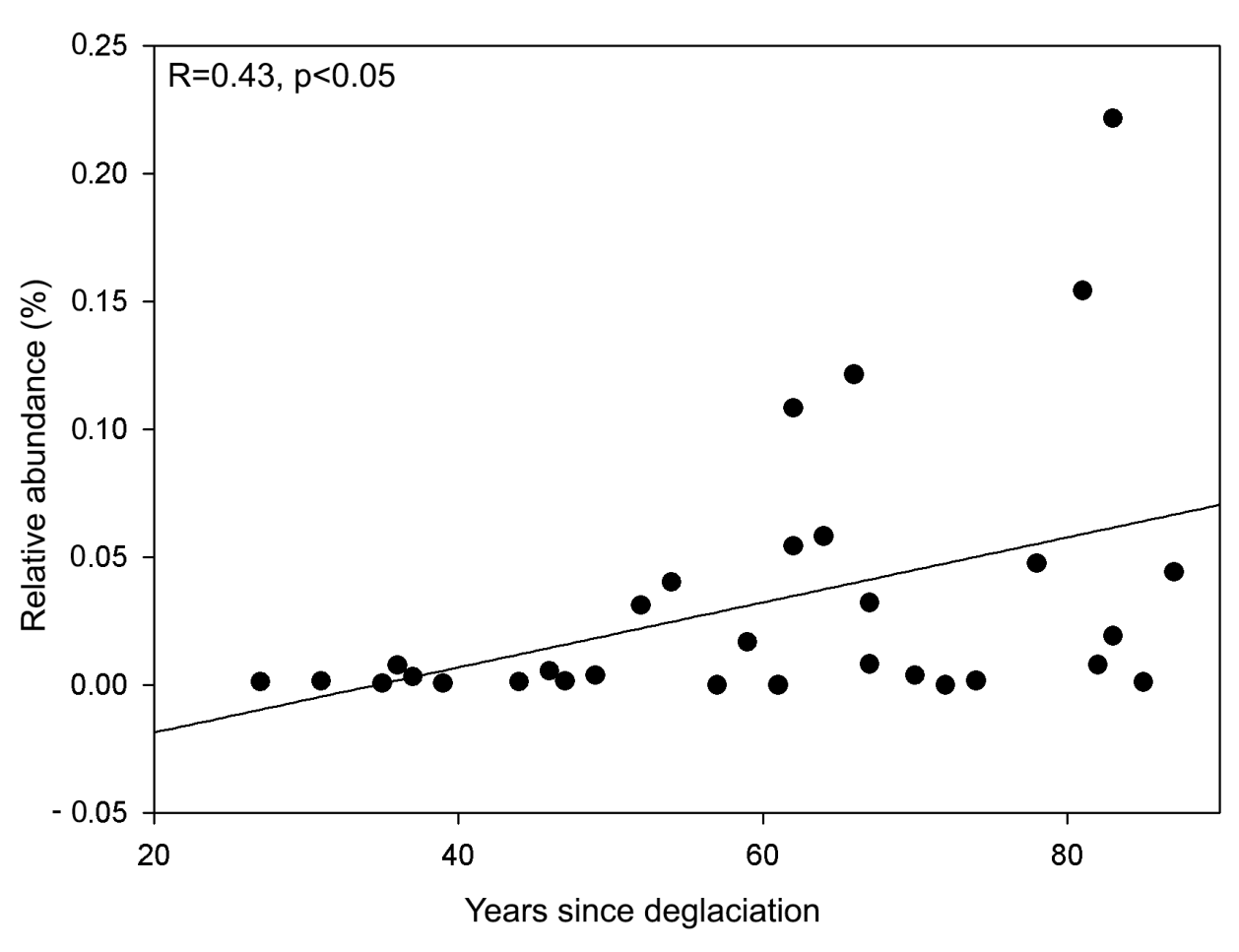


**Supplementary Fig. S1.** Relative abundance of ectomycorrhizal fungi showed a significant linear regression with age since deglaciation.


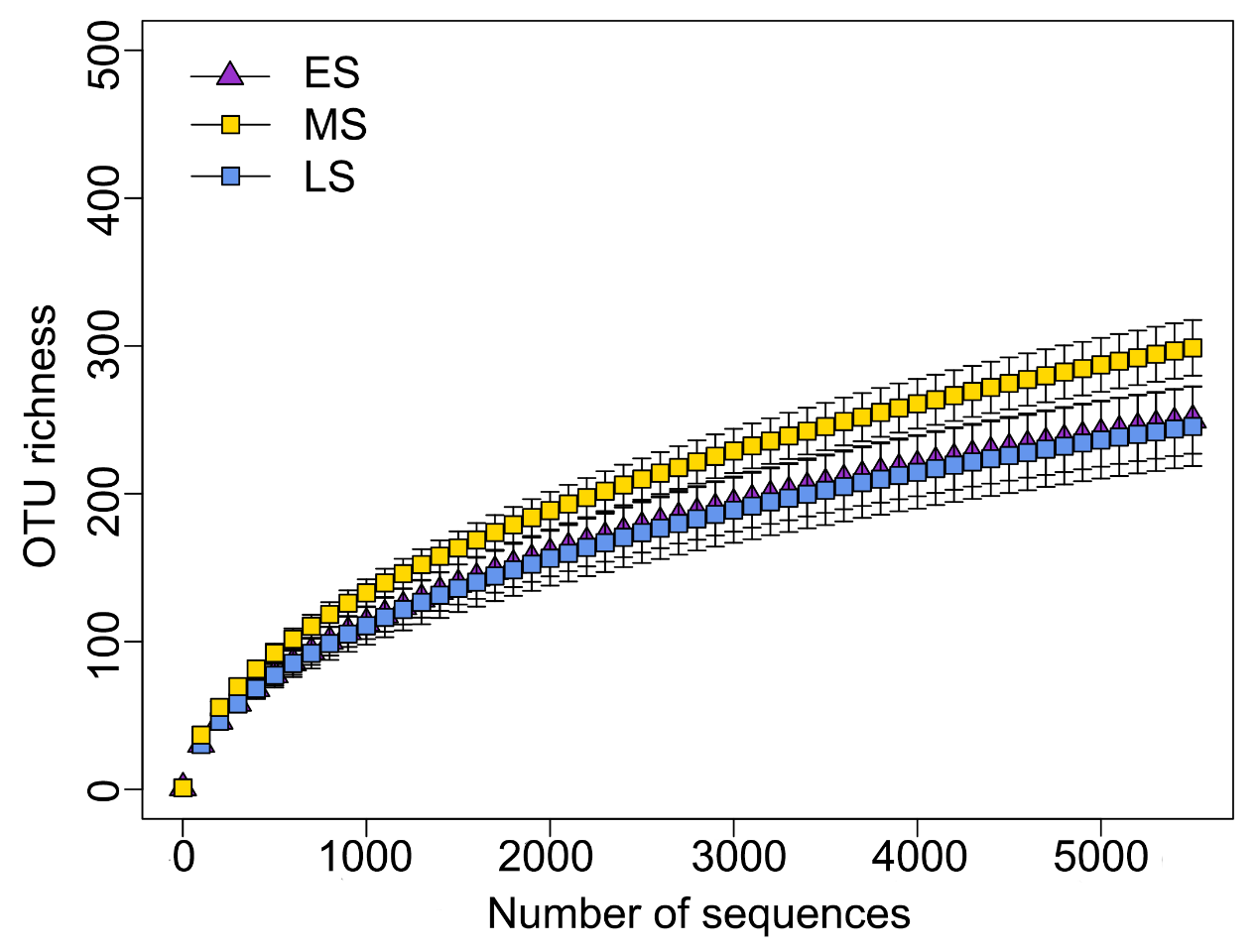


**Supplementary Fig. S2** Rarefaction curve for each stage based on fungal OTUs at a dissimilarity level of 3%. ES, Early Stage; MS, Mid Stage; LS, Late Stage. Mid Stage has significantly greater OTU richness compared to ES and LS (P=<0.05).


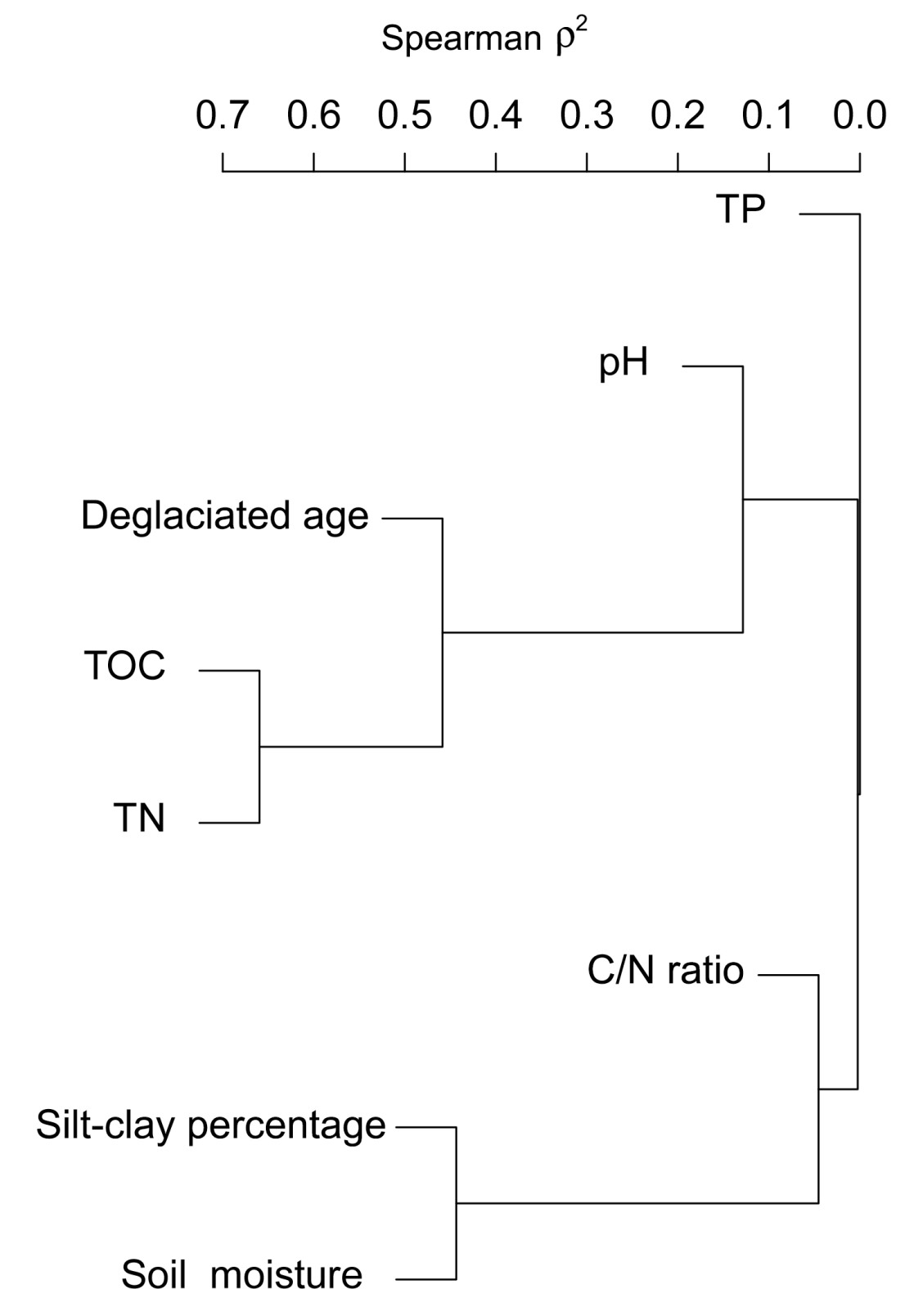


**Supplementary Fig. S3.** Cluster analysis of all measured environmental variables and age since deglaciation in the retreating glacier area. Abbreviations: TOC, Total Organic Carbon; TN: Total Nitrogen; C/N ratio: Carbon/Nitrogen ratio. Total of percentage silt and clay content are used to indicate soil texture here.

**
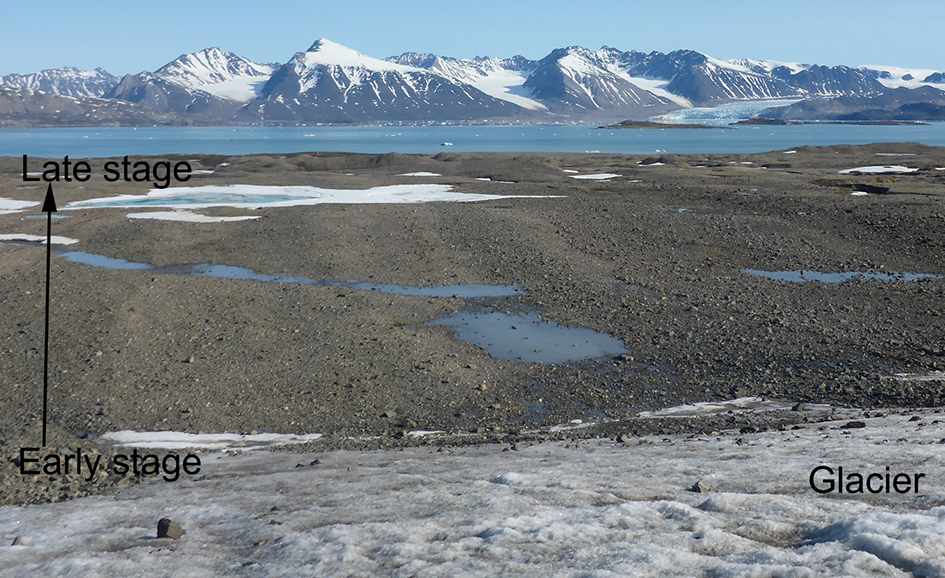
**

**Supplementary Fig. S4** Photos of sampling sites in Midre Lovénbreen glacier showing showing soil stages.

**
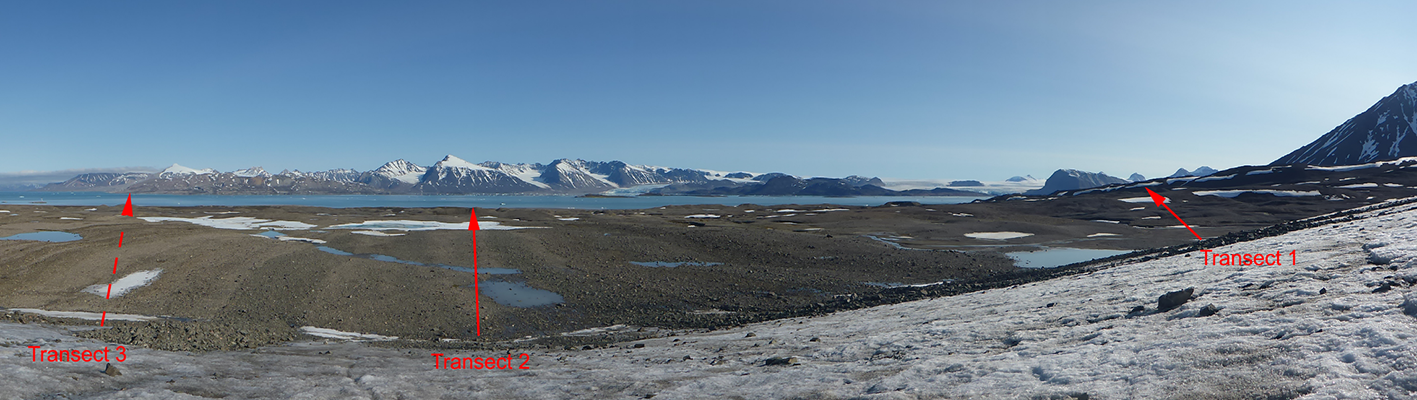
**

**Supplementary Fig. S5** Photos of sampling sites in Midre Lovénbreen glacier showing three sampling transects.

**
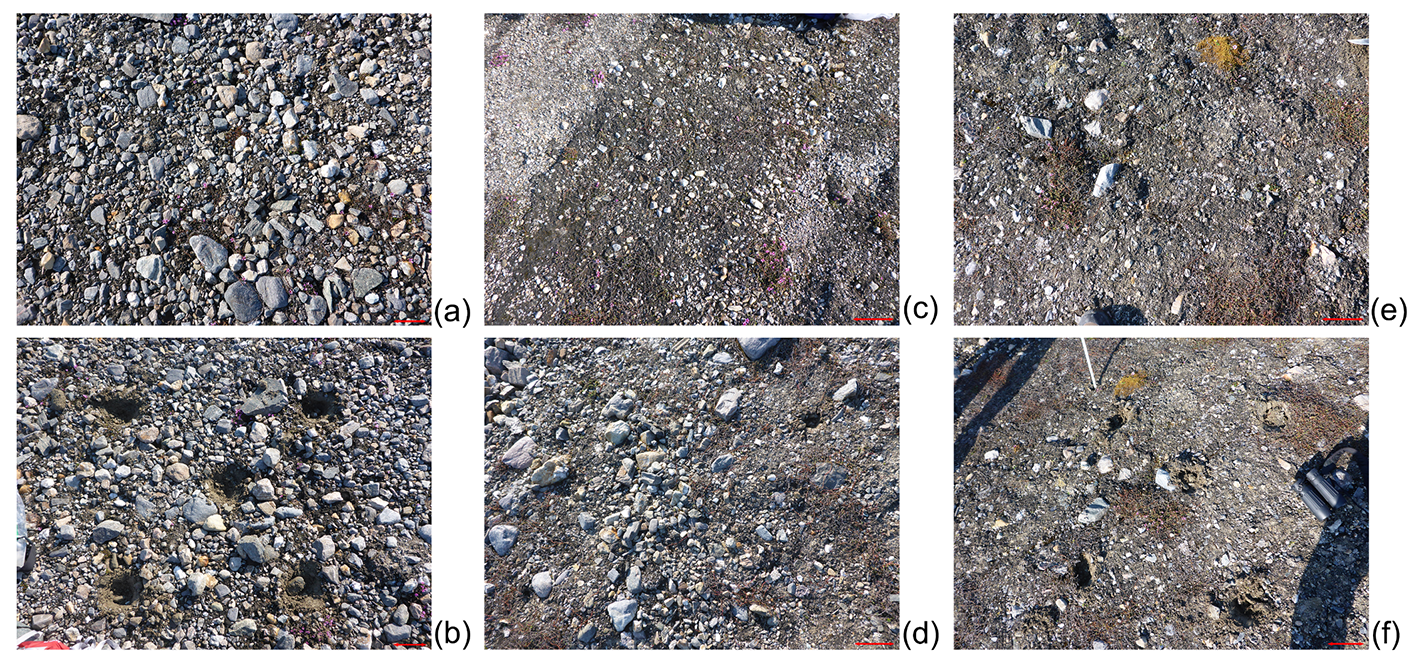
**

**Supplementary Fig. S6** Photos of sampling sites in Midre Lovénbreen glacier showing soils of three stages. (a)(b), early stage; (c)(d), mid stage; (e)(f), late stage.

**Supplementary Table 1** 77 OTUs as indicator OTUs from three soil developing stage. Only OTUs with greater than 10% frequency and an indicator value >0.3 and a p value <0.05 was selected as indicator OTUs. ES, Early Stage; MS, Mid Stage; LS, Late Stage.

| OTUs | Classification | Soil stage | Reads | Indval value | p value | Frequence |
| --- | --- | --- | --- | --- | --- | --- |
| Otu00037 | Ascomycota | ES | 6704 | 0.81 | 0.023 | 100% |
| Otu00046 | Ascomycota | ES | 3709 | 0.71 | 0.005 | 97% |
| Otu00131 | Ascomycota | ES | 2857 | 0.68 | 0.023 | 42% |
| Otu00423 | Ascomycota | LS | 1433 | 0.50 | 0.012 | 13% |
| Otu00433 | Basidiomycota | LS | 1296 | 0.70 | 0.042 | 68% |
| Otu00480 | Ascomycota | MS | 1233 | 0.84 | 0.005 | 61% |
| Otu00629 | Ascomycota | ES | 1117 | 0.69 | 0.042 | 81% |
| Otu00722 | Ascomycota | LS | 294 | 0.50 | 0.013 | 23% |
| Otu00835 | Ascomycota | MS | 878 | 0.68 | 0.025 | 77% |
| Otu00863 | Ascomycota | ES | 879 | 0.66 | 0.006 | 42% |
| Otu00887 | Ascomycota | ES | 887 | 0.68 | 0.001 | 84% |
| Otu01436 | Ascomycota | MS | 549 | 0.65 | 0.046 | 58% |
| Otu01460 | Ascomycota | ES | 546 | 0.71 | 0.039 | 77% |
| Otu01510 | unclassified | ES | 513 | 0.69 | 0.006 | 29% |
| Otu01687 | Ascomycota | MS | 472 | 0.65 | 0.023 | 42% |
| Otu01689 | Ascomycota | LS | 416 | 0.61 | 0.025 | 48% |
| Otu01747 | Ascomycota | ES | 452 | 0.71 | 0.017 | 68% |
| Otu01861 | Ascomycota | MS | 371 | 0.71 | 0.005 | 61% |
| Otu01990 | Ascomycota | ES | 331 | 0.63 | 0.027 | 55% |
| Otu02040 | unclassified | LS | 321 | 0.49 | 0.012 | 19% |
| Otu02156 | Ascomycota | MS | 259 | 0.55 | 0.045 | 52% |
| Otu02207 | Ascomycota | LS | 286 | 0.57 | 0.035 | 52% |
| Otu02217 | Zygomycota | ES | 269 | 0.56 | 0.045 | 52% |
| Otu02292 | Ascomycota | ES | 256 | 0.73 | 0.007 | 65% |
| Otu02363 | unclassified | LS | 261 | 0.83 | 0.001 | 19% |
| Otu02971 | Ascomycota | LS | 158 | 0.45 | 0.036 | 19% |
| Otu02993 | unclassified | MS | 193 | 0.53 | 0.037 | 48% |
| Otu03313 | Ascomycota | ES | 180 | 0.56 | 0.043 | 52% |
| Otu03517 | Ascomycota | ES | 154 | 0.58 | 0.044 | 61% |
| Otu03538 | Ascomycota | ES | 167 | 0.62 | 0.005 | 52% |
| Otu03789 | unclassified | MS | 144 | 0.53 | 0.043 | 48% |
| Otu03819 | Ascomycota | LS | 113 | 0.46 | 0.041 | 35% |
| Otu04105 | Ascomycota | ES | 114 | 0.51 | 0.051 | 55% |
| Otu04159 | Ascomycota | ES | 125 | 0.70 | 0.006 | 61% |
| Otu04376 | Ascomycota | LS | 106 | 0.61 | 0.005 | 26% |
| Otu04671 | Ascomycota | ES | 117 | 0.46 | 0.027 | 23% |
| Otu05159 | Ascomycota | MS | 103 | 0.47 | 0.029 | 23% |
| Otu05473 | unclassified | ES | 97 | 0.55 | 0.029 | 35% |
| Otu05757 | Ascomycota | LS | 81 | 0.38 | 0.049 | 23% |
| Otu05888 | unclassified | MS | 86 | 0.56 | 0.02 | 48% |
| Otu06112 | Ascomycota | ES | 83 | 0.47 | 0.037 | 45% |
| Otu06192 | Zygomycota | ES | 74 | 0.73 | 0.004 | 35% |
| Otu06202 | Basidiomycota | LS | 82 | 0.39 | 0.024 | 19% |
| Otu06422 | Ascomycota | ES | 79 | 0.59 | 0.013 | 48% |
| Otu06699 | Ascomycota | LS | 50 | 0.40 | 0.025 | 16% |
| Otu07213 | Ascomycota | MS | 67 | 0.51 | 0.015 | 29% |
| Otu07382 | Ascomycota | ES | 64 | 0.52 | 0.014 | 39% |
| Otu07412 | Ascomycota | LS | 58 | 0.44 | 0.028 | 19% |
| Otu07429 | Ascomycota | MS | 48 | 0.48 | 0.033 | 32% |
| Otu07565 | unclassified | ES | 59 | 0.54 | 0.011 | 26% |
| Otu07681 | Ascomycota | ES | 55 | 0.50 | 0.028 | 35% |
| Otu07702 | Ascomycota | MS | 34 | 0.40 | 0.046 | 19% |
| Otu08310 | unclassified | ES | 51 | 0.48 | 0.025 | 32% |
| Otu08765 | unclassified | MS | 44 | 0.44 | 0.034 | 26% |
| Otu09186 | Zygomycota | ES | 42 | 0.45 | 0.016 | 26% |
| Otu09461 | Ascomycota | LS | 41 | 0.50 | 0.006 | 10% |
| Otu09767 | unclassified | ES | 37 | 0.51 | 0.036 | 29% |
| Otu09986 | Ascomycota | MS | 35 | 0.38 | 0.034 | 23% |
| Otu10071 | Ascomycota | ES | 36 | 0.66 | 0.002 | 29% |
| Otu10858 | Ascomycota | ES | 31 | 0.36 | 0.036 | 16% |
| Otu12169 | Ascomycota | ES | 23 | 0.35 | 0.03 | 16% |
| Otu12531 | unclassified | ES | 22 | 0.40 | 0.021 | 13% |
| Otu12995 | unclassified | ES | 20 | 0.44 | 0.02 | 26% |
| Otu13425 | unclassified | MS | 19 | 0.43 | 0.026 | 26% |
| Otu13459 | unclassified | ES | 17 | 0.37 | 0.046 | 23% |
| Otu13800 | Ascomycota | LS | 10 | 0.30 | 0.035 | 13% |
| Otu14155 | Ascomycota | ES | 17 | 0.58 | 0.003 | 23% |
| Otu14213 | unclassified | ES | 17 | 0.61 | 0.001 | 32% |
| Otu15855 | unclassified | LS | 14 | 0.65 | 0.003 | 16% |
| Otu16047 | unclassified | ES | 13 | 0.35 | 0.048 | 16% |
| Otu16163 | unclassified | ES | 14 | 0.40 | 0.014 | 13% |
| Otu17694 | Ascomycota | ES | 12 | 0.40 | 0.019 | 13% |
| Otu18266 | unclassified | ES | 11 | 0.38 | 0.035 | 16% |
| Otu19265 | unclassified | LS | 10 | 0.37 | 0.041 | 16% |
| Otu19663 | Ascomycota | MS | 10 | 0.33 | 0.054 | 16% |
| Otu19892 | Basidiomycota | LS | 10 | 0.30 | 0.045 | 10% |
| Otu20737 | unclassified | MS | 8 | 0.40 | 0.028 | 19% |

**Supplementary Table 2** The sites sampled in this study. GPS coordinate was used to label the sampling site of each sample. The deglaciated age of each site is interpreted by isochrones.

| Sample name | N | W | Deglaciated age |
| --- | --- | --- | --- |
| KOPRI 303 | 78°53′47″ | 12°02′51″ | 0 |
| KOPRI 6 | 78°54′20″ | 12°04′36″ | 83 |
| KOPRI 20 | 78°54′18″ | 12°04′45″ | 81 |
| KOPRI 47 | 78°54′14″ | 12°04′41″ | 74 |
| KOPRI 62 | 78°54′12″ | 12°04′24″ | 70 |
| KOPRI 103 | 78°54′08″ | 12°04′16″ | 64 |
| KOPRI 120 | 78°54′06″ | 12°04′22″ | 61 |
| KOPRI 132 | 78°54′05″ | 12°04′26″ | 59 |
| KOPRI 191 | 78°53′58″ | 12°03′53″ | 46 |
| GFL1_1 | 78°53′32″ | 12°04′29″ | 0 |
| GFL 1_2 | 78°53′34″ | 12°04′37″ | 14 |
| GFL 1_3 | 78°53′35″ | 12°04′43″ | 19 |
| GFL 1_4 | 78°53′36″ | 12°04′52″ | 23 |
| GFL 1_5 | 78°53′37″ | 12°04′56″ | 27 |
| GFL 1_6 | 78°53′38″ | 12°04′59″ | 31 |
| GFL 1_7 | 78°53′40″ | 12°04′59″ | 35 |
| GFL 1_8 | 78°53′41″ | 12°04′60″ | 37 |
| GFL 1_9 | 78°53′44″ | 12°05′01″ | 39 |
| GFL 1_10 | 78°53′45″ | 12°05′10″ | 44 |
| GFL 1_11 | 78°53′46″ | 12°05′20″ | 49 |
| GFL 1_12 | 78°53′48″ | 12°05′27″ | 54 |
| GFL 1_13 | 78°53′50″ | 12°05′40″ | 62 |
| GFL 1_14 | 78°53′52″ | 12°05′45″ | 66 |
| GFL 1_15 | 78°53′54″ | 12°05′57″ | 67 |
| GFL 2_1 | 78°53′46″ | 12°03′34″ | 0 |
| GFL 2_2 | 78°53′49″ | 12°03′41″ | 20 |
| GFL 2_3 | 78°53′52″ | 12°03′49″ | 25 |
| GFL 2_4 | 78°53′55″ | 12°03′53″ | 36 |
| GFL 2_5 | 78°53′58″ | 12°03′59″ | 47 |
| GFL 2_6 | 78°54′00″ | 12°04′11″ | 52 |
| GFL 2_7 | 78°54′03″ | 12°04′20″ | 57 |
| GFL 2_8 | 78°54′07″ | 12°04′23″ | 62 |
| GFL 2_9 | 78°54′10″ | 12°04′25″ | 67 |
| GFL 2_10 | 78°54′14″ | 12°04′30″ | 72 |
| GFL 2_11 | 78°54′17″ | 12°04′35″ | 78 |
| GFL 2_12 | 78°54′20″ | 12°04′17″ | 82 |
| GFL 2_13 | 78°54′20″ | 12°04′33″ | 83 |
| GFL 2_14 | 78°54′22″ | 12°04′25″ | 85 |
| GFL 2_15 | 78°54′24″ | 12°04′31″ | 87 |

**Supplementary Table 3** A packed matrix order categorizing nestedness of each sample from high to low. Deglaciated age and OTU richness are listed together. Five samples (highlighted) among the first six samples are from the mid stage.

| Packed matrix order | Sample name | Deglaciated age | OTU richness |
| --- | --- | --- | --- |
| 1 | KOPRI103 | 64 | 250 |
| 2 | 1_15 | 67 | 224 |
| 3 | 2_6 | 52 | 207 |
| 4 | KOPRI62 | 70 | 229 |
| 5 | 2_15 | 87 | 181 |
| 6 | 1_13 | 62 | 208 |
| 7 | 2_4 | 36 | 169 |
| 8 | KOPRI191 | 46 | 191 |
| 9 | 1_11 | 49 | 185 |
| 10 | 1-12 | 54 | 219 |
| 11 | 2_8 | 62 | 198 |
| 12 | 1_8 | 37 | 194 |
| 13 | 2_12 | 82 | 173 |
| 14 | 2_14 | 85 | 175 |
| 15 | 1_7 | 35 | 170 |
| 16 | 2_9 | 67 | 164 |
| 17 | KOPRI132 | 59 | 166 |
| 18 | 1_10 | 44 | 164 |
| 19 | 1_14 | 66 | 151 |
| 20 | 1_6 | 31 | 144 |
| 21 | 2_5 | 47 | 149 |
| 22 | 2_13 | 83 | 137 |
| 23 | KOPRI47 | 74 | 134 |
| 24 | KOPRI120 | 61 | 138 |
| 25 | 2_7 | 57 | 136 |
| 26 | 2_10 | 72 | 118 |
| 27 | KOPRI20 | 81 | 114 |
| 28 | 2_11 | 78 | 117 |
| 29 | 1_9 | 39 | 99 |
| 30 | KOPRI6 | 83 | 113 |
| 31 | 1_5 | 27 | 90 |
